# Authors' reply: Re: Koga *et al*. Retrieval‐augmented generation versus document‐grounded generation: a key distinction in large language models

**DOI:** 10.1002/2056-4538.70013

**Published:** 2025-01-21

**Authors:** Katherine J Hewitt, Isabella C Wiest, Jakob N Kather

**Affiliations:** ^1^ Else Kroener Fresenius Center for Digital Health Faculty of Medicine and University Hospital Carl Gustav Carus, TUD Dresden University of Technology Dresden Germany; ^2^ Department of Medicine II, Medical Faculty Mannheim Heidelberg University Mannheim Germany; ^3^ Department of Medicine I Faculty of Medicine and University Hospital Carl Gustav Carus, TUD Dresden University of Technology Dresden Germany; ^4^ Medical Oncology, National Center for Tumor Diseases University Hospital Heidelberg Heidelberg Germany

We thank Koga *et al* for their knowledgeable comments on our work. Their letter highlights a valid question that requires clarification [1].

Our study assessed the ability of three large language models (LLMs) to diagnose neuropathology cases from free‐text descriptions of adult‐type diffuse gliomas, for which we compared two methodologies. The first method provided each model with the free‐text tumor descriptions alone, while the second approach additionally provided the models with a Word document of the WHO CNS5. We termed these approaches zero‐shot and retrieval‐augmented generation (RAG), respectively [2]. Koga *et al* point out that the methodology we describe in our paper as RAG, may be better described as document‐grounded generation, or in‐context learning.

While we agree with the definition of RAG provided in the letter as it was initially defined [3], the field has evolved significantly since the approach was first proposed by Lewis *et al* in 2020. Three paradigms of RAG are now increasingly recognized: naive RAG, advanced RAG, and modular RAG [4]. Naive RAG is an approach where the data for indexing are generally obtained offline and converted into a format such as PDF or Word, and uploaded with the query via the context window. Advanced RAG and modular RAG offer specific improvements to address the limitations of naive RAG; however, to achieve this, they utilize more technical approaches.

The intention for our paper was to use naive RAG. We chose this approach as it leverages the easiest possible way for improving an LLM response that would be reproducible by doctors, considering that most doctors would be unable to utilize the application programming interface and programmatically build a RAG pipeline. As discussed by Koga *et al*, the key difference between naive RAG and document‐grounding lies in how the document is utilized when the model retrieves its response [5]. Document‐grounding submits the document with the user query and is equivalent to inserting the entire document text into the context window [5]. Whereas with naive RAG, relevant parts of the document are identified by the model and used with the query to dynamically search its database [4]. Both approaches are examples of in‐context learning as they acquire additional knowledge from the prompt without requiring parameter updates [6].

Bereft of transparency from the LLM providers regarding how they process the document once it has been submitted via the graphical user interface, it is difficult to know whether naive RAG or document‐grounding was used to formulate a response. To our knowledge, details regarding how appended documents are utilized during a query are not freely available online by ChatGPT, Llama, or Claude. Furthermore, due to the speed of development in the field, technical aspects of how documents are utilized may have changed since our experiments were conducted earlier this year. Nonetheless, we contacted ChatGPT, Anthropic, and Poe for assistance in clarifying this point. All three providers confirmed that documents uploaded with a query are used for RAG. However, the responses from ChatGPT and Anthropic were both generated by bots, demonstrating the need for more reliable and greater transparency about the actual technical methods used.

We are grateful for your endorsement of our conclusions and appreciate the opportunity to address this important distinction. Further clarification and transparency are needed to definitively distinguish the mechanisms employed by specific LLM platforms, particularly regarding how appended documents are processed. We proffer that our work uses in‐context learning, as both RAG and document‐grounding are methods of this broader paradigm. Nevertheless, we remain committed to clarifying this matter and thank Koga *et al* for their engagement and valuable input.

## Author contributions statement

KJH wrote the first draft; review and critique; and final approval. ICW review and critique; and final approval. JNK review and critique; and final approval.
